# Novel MRPS9-ALK Fusion Mutation in a Lung Adenocarcinoma Patient: A Case Report

**DOI:** 10.3389/fonc.2021.670907

**Published:** 2021-06-08

**Authors:** Huamiao Zhou, Binyue Xu, Jili Xu, Guomeng Zhu, Yong Guo

**Affiliations:** ^1^ Department of Oncology, Zhejiang Provincial Hospital of Traditional Chinese Medicine, Hangzhou, China; ^2^ The First School of Clinical Medicine, Zhejiang Chinese Medical University, Hangzhou, China

**Keywords:** NSCLC, MRPS9-ALK, NGS, crizotinib, ALK-TKI

## Abstract

Anaplastic lymphoma kinase (ALK) rearrangements account for approximately 5–6% of non–small-cell lung cancer (NSCLC) patients. In this study, a case of lung adenocarcinoma harboring a novel MRPS9-ALK fusion is reported. The patient responded well to the first and second generation of ALK-tyrosine kinase inhibitors (ALK-TKIs) (crizotinib then alectinib), as her imaging findings and clinical symptoms significantly improved. At last follow-up, over 21 months of overall survival (OS) has been achieved since ALK-TKI treatment. The progression-free survival (PFS) is already ten months since alectinib. The adverse effects were manageable. The case presented here provides first clinical evidence of the efficacy of ALK-TKIs in NSCLC patients with MRPS9-ALK fusion.

## Introduction

Lung cancer is one of the most prevalent cancers and is currently the leading cause of cancer-related mortality worldwide (1). Non-small-cell lung cancer (NSCLC), epithelial in origin, accounts for 80% of all lung cancers, including adenocarcinoma, squamous cell carcinoma, and large cell carcinoma, among which adenocarcinoma is the most common pathological subtype (2). Anaplastic lymphoma kinase (ALK) was first identified in anaplastic large-cell lymphoma (ALCL) in 1994 as a fusion gene, which is a transmembrane receptor tyrosine kinase, as well as a member of the insulin receptor superfamily (3). ALK gene rearrangements have been reported in 5-6% of NSCLC patients, especially in mild or non-smokers (4). The echinoderm microtubule-associated like four (EML4)–ALK fusion is the most common fusion and was first reported in 2007 (5). Over 90 fusion partners of the ALK gene have been discovered to date in NSCLC (6). The discovery of the driver mutation in lung adenocarcinoma provides new strategies and promise for the treatment of unresectable tumors. Crizotinib, an aminopyridine compound, as the first ALK tyrosine kinase inhibitor (TKI), was approved for the treatment of ALK‐positive NSCLC in 2011 (7). In addition, it was rapidly followed by the ALK-TKIs of second-generation (alectinib, ceritinib, and brigatinib) and third-generation (lorlatinib).

However, it remains unclear whether the resulting protein structures translated from fused ALK transcripts retain the entire kinase domain of ALK, and will thus respond to ALK-TKI therapy. In addition, how the different fusion partners affect the responsiveness to targeted therapies and acquired resistance is still under investigation. And accurate identification of the ALK gene status is thus crucial for selecting the appropriate therapy. Based on this, a combination of different technologies, such as fluorescence *in situ* hybridization (FISH), immunohistochemistry (IHC), and next-generation sequencing (NGS), might be necessary to explore the ALK status and provide better clinical decision-making (8, 9). In this report, for the first time an unreported MRPS9-ALK fusion mutation in a lung adenocarcinoma patient is presented by using targeted next generation sequencing (NGS). And the patient rapidly responded to treatment with ALK-TKI with remarkable therapeutic effect.

## Case Presentation

**Figure 1:**
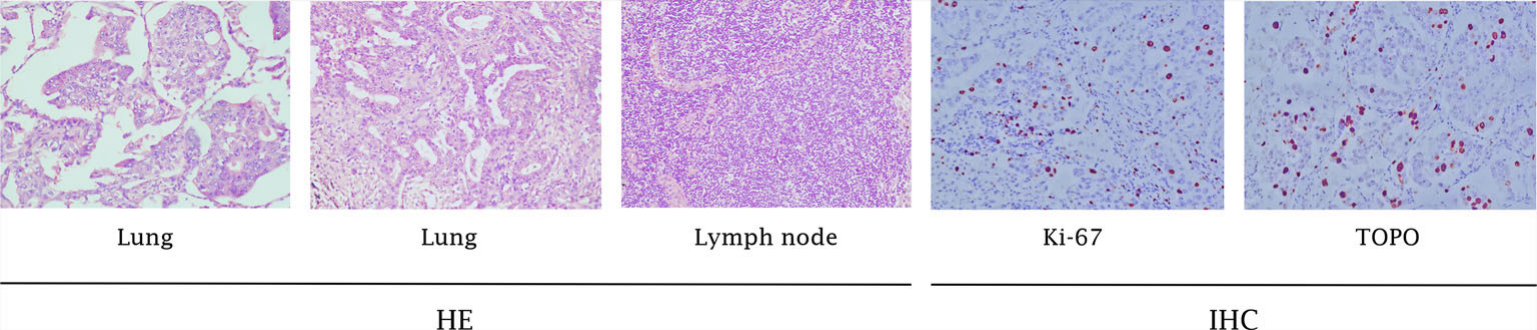
Pathology results. H&E staining of the biopsy specimen showed poorly-differentiated NSCLC. IHC staining showed positive expression of Ki-67 and TopoII (x100).

During a regular health check in July 2013, a 60-year-old non-smoking Chinese woman was diagnosed with lung carcinoma. A mass in the right upper lobe and an enlarged lymph node in the right hilar were showed in thoracic computed tomography (CT). Then, she received a video-assisted thoracic surgery (VATS)-assisted right upper lobectomy. A pulmonary nodule (2.0 cm × 1.0 cm × 1.0 cm) was surgically removed. Postoperative pathology confirmed that it was a stage IA (pT1N0M0, 7^th^ UICC/AJCC) poorly-differentiated adenocarcinoma. The immunohistochemistry (IHC) analysis showed a positive expression of Napsin A, TTF-1, and Ki-67, but a negative expression of ALK and K-RAS (**Figure 1**). The epidermal growth factor receptor (EGFR) mutation was not detected by using fluorescent polymerase chain reaction (PCR). Due to the early pathological TNM stage and negative margin (R0), the patient did not undergo adjuvant therapy; only regular follow-up and observation were required (NCCN Guidelines, 2013). However, 16 months after surgery, the patient suffered from intermittent headache, right limb numbness, and weakness. Cranial magnetic resonance imaging (MRI) suggested a metastatic tumor of the left parietal lobe. Therefore, she was treated with whole-brain radiotherapy (3500cGy/14F) for brain metastasis (since December 11, 2014) and four cycles of TP regimen (paclitaxel + cisplatin) chemotherapy (since January 30, 2015). During therapy, she developed a headache, asthenia, poor appetite, and grade 1 nausea and vomiting, which were gradually relieved after symptomatic treatment of dehydration, lowering the intracranial pressure, antiemetics, and gastro-protection. Grade 1 myelosuppression was also observed after chemotherapy. At the end of treatment, the patient achieved a partial response (PR) based on the imaging findings.

Progression of the disease was observed again in August, 2018. A thoracic CT showed small nodules on both lungs with enlarged mediastinal lymph nodes, which were considered to be metastatic foci. The cranial MR also showed new lesions in the right frontal lobe, right occipital lobe, and left parietal lobe. The patient experienced recurrent coughing and expectoration. She was treated with a PC regimen (pemetrexed + carboplatin) chemotherapy for two cycles (since August 28, 2018). Because of the poor therapeutic efficacy, the treatment regimen was changed to the PC regimen plus bevacizumab 400 mg targeted therapy for four cycles. The efficacy was evaluated as stable disease (SD). In addition, a next-generation sequencing (NGS) analysis was performed on the mediastinal puncture tissue based on a 425-gene panel targeting eight genes (EGFR, ALK, RET, POS1, MET, ERBB2, KRAS, and BRAF) (GENESEEQ Technology Inc, Nanjing, China). A rare novel ALK fusion (IGR (upstream MRPS9)~ALK) (**Figure 3A**) was found using the NGS assay, in which the intact kinase domain of ALK was retained. The diagram of the MRPS9-ALK fusion is displayed in **Figure 3B**. In addition, a frameshift mutation in exon 2 of RET (c.198delC; p.F66fs) was detected. EGFR mutation and ROS-1 rearrangement were not detected. Due to the uncertainty efficacy of crizotinib on the atypical mutation of ALK and for economic concerns (crizotinib was not covered by Chinese health insurance until Oct. 2018), the patient was not treated with ALK-TKI immediately but was treated with pemetrexed and bevacizumab as a maintenance therapy for six cycles (from February 2019 to July 2019).

**Figure 2:**
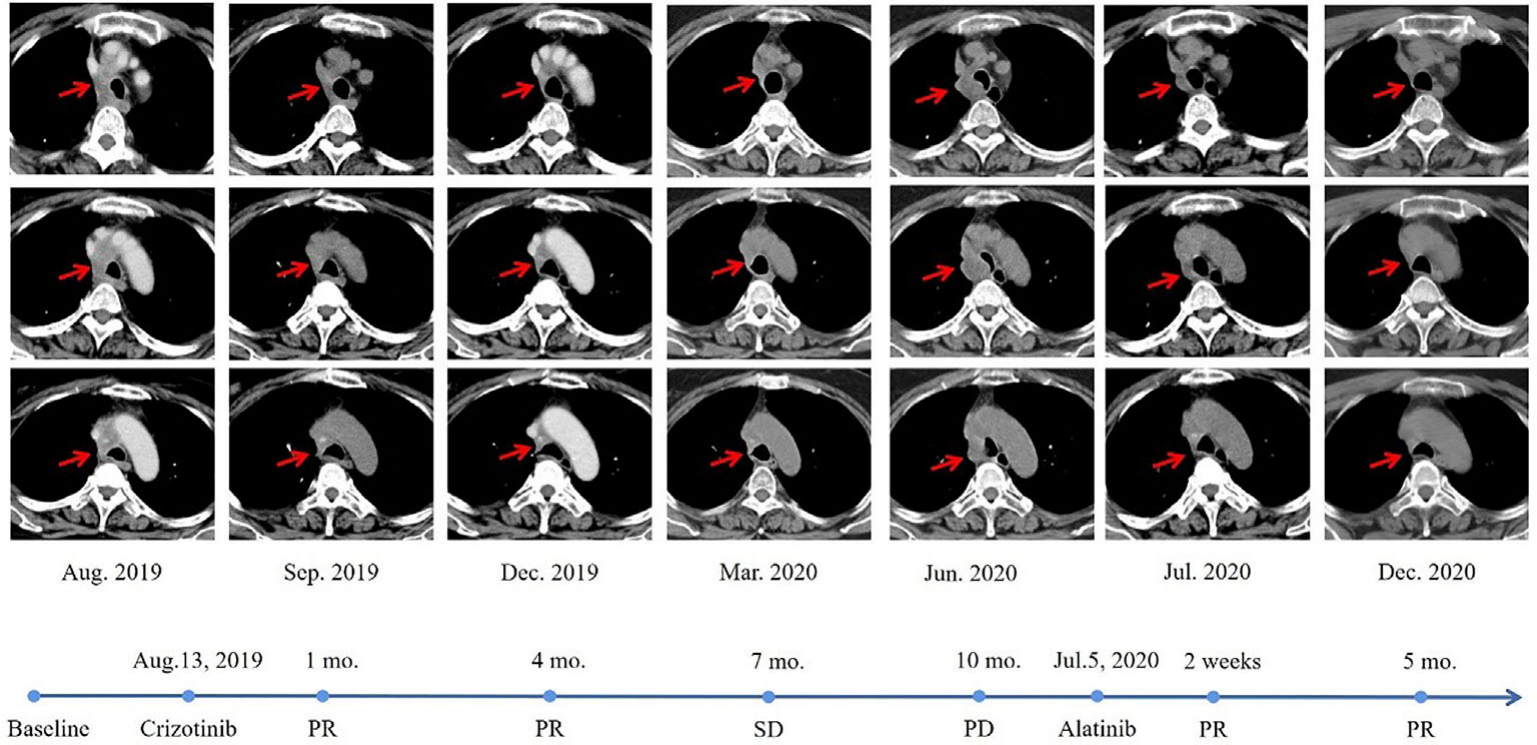
Radiological evaluation before and after therapy. The CT scan images demonstrated a significant reduction in pulmonary lesions.

Unfortunately, during the period of maintenance therapy, she felt mild pain in the left lower extremity. On May 14, 2019, the MRI of the left femur showed an abnormal signal shadow in the upper left femur, which indicated tumor metastasis. Zoledronic acid was used to prevent bone destruction immediately (since May 16, 2019). Considering the continuous progression of the disease and the patient’s poor physical condition, she consented to take crizotinib at 250mg orally twice daily since August 13, 2019. Luckily, a reassessment demonstrated that crizotinib had a significant effect on the patient (**Figure 2**). Nevertheless, after 10 months of therapy with crizotinib, the disease progressed again according to the thoracic CT on June 13, 2020, which showed enlarged lymph nodes adjacent to the right main trachea, compressing the airway. A cranial MRI also showed new intracranial lesions. The patient soon presented with progressive shortness of breath. To clarify the pathological type and genetic landscape of the recurrent tumor, a biopsy was recommended by the medics. However, the patient refused because of the high cost. In the light of the NGS results and clinical guidelines, she started to take 600 mg of alectinib orally twice daily on July 5, 2020, and her clinical symptoms significantly improved within 3 days of commencing treatment. The patient maintained stable disease on the alectinib treatment, with a PFS time of over ten months at last follow-up (**Figure 2**).

## Discussion

**Figure 3:**
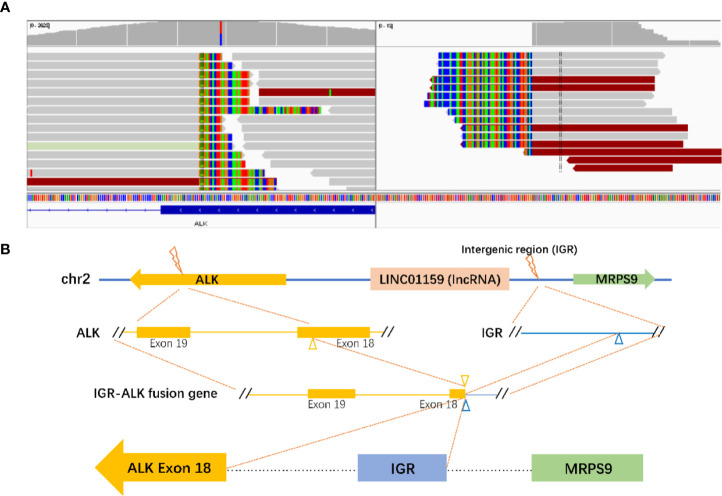
Identification of the MRPS9-ALK fusion. **(A)** Sequencing reads of ALK and LINCO1159 & MRPS9 were visualized using the Integrative Genomics Viewer (IGV). **(B)** The structure schematic map of the MRPS9-ALK fusion locus. Intergenic region (IGR) of LINCO1159 & MRPS9 were fused to exon 18 of the ALK (yellow).

Based on literature retrieval, this is the first report to demonstrate the presence of a novel MRPS9-ALK mutation in NSCLC. The product may consist of the intergenic region of the LINCO1159 and MRPS9 genes (upstream MRPS9) and the 18 exon of the ALK gene (**Figure 3**). A frameshift mutation in exon 2 of RET was also detected using NGS in this case. Frameshift mutations are certain base deletions or insertions within the gene coding region that, disturb the reading frame and thus alter the downstream code (10). Frameshift mutations of tumor suppressor genes always result in either silent or conservative changes, which lead to cancer development. For example, UVRAG, a tumor suppressor, when truncated by a frameshift mutation can switch to an oncogene in colorectal cancer (11). However, the specific significance of a frameshift mutation in the oncogene, RET, remains to be further studied.

Previous work has demonstrated that the ALK fusion protein can form homodimers (or oligomers) through the N-terminal dimerization sites of the ALK fusion protein, which is a mechanism mimics ligand bindings (12). This phenomenon leads to the activation of the ALK catalytic domain, that is, the autophosphorylation of the ALK tyrosine kinase domain (13). Consequently, common characteristics of ALK fusions have been proposed that, also result in their carcinogenicity: 1) The entire tyrosine kinase domain is contained in the fusion protein (typically at exon 20) when the conserved breakpoint in the ALK gene occurs; 2) A promoter derived from the N-terminal fusion partner leads to the constitutive expression of the ALK fusion protein; and 3) The fusion partner must include an oligomerization domain (14). During the ALK rearrangement, the oligomerization domain of the fusion partner mediates the oligomerization of ALK fusion protein, which further induces the constitutive activation of the kinase, and thereby activates downstream ALK signaling events.

Despite the unclear way of gene integration and the specific significance, the function prediction in this case showed an intact ALK kinase domain in the fusion. Thus, this may result in the activation of ALK kinase. The application of crizotinib turned out to have a significant response as expected, suggesting that this novel fusion gene can activate the ALK autophosphorylation and trigger downstream signaling pathways, which further drive oncogenesis.

Crizotinib is an orally available small molecule competitive ATP inhibitor of ALK/MET/ROS1 tyrosine kinases (15) that, has shown a significantly longer PFS and higher overall response rate (ORR) compared to chemotherapy in considerable clinical studies (16, 17). However, the efficacy of crizotinib was noted to not be uniform across all fusions tested (18). Emerging data have also suggested a significant difference in the sensitivity and resistance of the clinical responsiveness to ALK TKIs among different ALK fusions (19). According to the characteristics of ALK fusion, one reasonable explanation is that different variations at the N-terminus of the ALK fusions affects its subcellular localization and the properties of resulting protein, such as stability and activity, and then further leads to the impact of ALK TKIs on downstream signaling. It has also been demonstrated that patients harboring complex ALK fusions (coexisting canonical and non-canonical ALK fusions) are associated with better treatment outcomes in ALK TKI therapy (20). For clinicians, to better serve clinical decision-making, it is vital to determine the mutational status of the ALK kinase domain, as well as the therapeutic implications of the partner involved in the fusion.

The patient’s disease worsened again after 10 months of crizotinib application. It has been reported that the rapid development of resistance and the consequent tumor relapse within one to two years was a major limitation of crizotinib treatment (21). The central nervous system (CNS) is a frequent site of relapse on crizotinib, since the limited penetration of crizotinib into the CNS. In this case, the progression of the metastatic brain tumor was seen 10 months after crizotinib treatment. Mutations in the ALK tyrosine kinase domain and the ALK gene amplification are potential mechanisms of resistance (22). Alectinib, as a first-line therapy for patients with ALK-positive NSCLC approved in November 2017, has advantages over crizotinib and is the best candidate for patients with CNS metastases. Alectinib was reported not a substrate of P-glycoprotein (P-gp), that is a key efflux transporter in blood–brain barrier penetration. This enable alectinib cross the blood-brain barrier in appreciable quantities (23). Measurable concentrations of alectinib in the cerebrospinal fluid (CSF) and the linear relation between paired CSF and plasma concentrations also demonstrate the penetration of alectinib into the CNS (24). A liquid biopsy (LB) may be the first choice in detecting resistance mechanisms and thus guiding the subsequent therapy, since it is noninvasive, painless, and can be repeated over time compared to tissue biopsy (25, 26). However, the tumor burden and the metastatic sites, as well as the pre-analytical phase and the optimization of the different parameters, will significantly affect the quantity and quality of the nucleic acids obtained from a plasma sample (27). Therefore, irrespective of the potential, which cannot be underestimated, there are still some limitations of LB, which cannot be neglected.

Variant allele frequency (VAF) of ALK mutation detected in the case was 1.1%. VAF refers to the proportion of sequencing reads harboring the mutant gene to all sequencing reads at the gene site, which turns out to be a valid predictive and prognostic factor in patients with NSCLC according to the growing studies. High mutation abundance was reported to have a significant association with good response to EGFR-TKI treatment and consequent improved PFS (28, 29). Hence, the relative mutation abundance can predict responsiveness to TKI treatment. And moreover, dynamic monitoring of the mutation abundance during therapy contributes to exploring the mechanisms of resistance and predicting clinical outcomes. However, altered abundance may occur during disease progression and TKI and/or chemotherapy (30). The intratumor heterogeneity might add an extra level of difficulty to the application of mutation abundance as well, requiring detecting methods with higher sensitivity and sufficient sequencing coverage for VAF mutations capturing (31, 32). However, there are few studies investigating the effect of ALK VAF in NSCLC and the majority of existing studies are based on circulating tumor DNA (ctDNA), which may be not appropriate to compare with tissue biopsies. Although higher ALK VAF may suggest a better response of ALK-TKI treatment in theory, interestingly, correlation between ALK expression levels and alectinib penetration in the tumor was not observed early after the administration of single dose in neuroblastoma xenografts (33). Further investigation on revealing the correlation between VAF and high CNS penetrance of alectinib may benefit the better clinical application of alectinib at an individualized level (34).

In the era of personalized and precise therapy, the clinical significance of fusion gene detection has been gradually proven, and more specific subtyping of NSCLC is required for the selection of therapeutic options. Growing technical methods, including FISH, IHC, and NGS, have been applied to detect fusion variants. With the advantages of turnaround times and reduced cost, IHC is increasingly recommended as a screening method for ALK. Interestingly, NGS showed an opposite result to IHC in our case. There may be several possibilities for this difference. It is well known that the sensitivity and specificity of the method depend on multiple factors, such as the quantity and quality of the biological samples, the type or source of antibodies, the antigen retrieval, antibody detection, amplification techniques, and the technological platforms (35–38). In this case, the first surgical sample submitted for histopathologic diagnosis was stored in formalin-fixed paraffin-embedded (FFPE) tissue, and the DNA or RNA of which may have been substantially degraded. The low transcriptional activity of the promoter-enhancer region and relatively low ALK protein concentrations may further lead to a false-negative result in IHC (39). Furthermore, FISH and IHC may lead to false-negative results or missed identification of the target, as they cannot achieve the precise determination of the unknown fusion gene partner and the breakpoint, which results in discordant results between the two detection methods (40–42). In this situation, in order to drive the triage of cases for molecular testing, the clinical application of NGS should be taken adequately into consideration to improve the accuracy of target identification (43).

However, the discordance of ALK status among FISH, IHC, and NGS may also be associated with the existence of nonproductive rearrangements, which may be due to the unknown biological mechanisms of biological complexity involved in transcriptional or post-transcriptional processes. Ying, J et al. indicated that some uncanonical ALK fusions detected using DNA NGS did not generate aberrant transcripts or proteins (44). This finding suggested that the potential unreliability of DNA NGS in identifying the uncommon ALK genomic breakpoint for predicting the efficacy of matched targeted therapy in NSCLC. X. Du et al. reported an interesting case that was ALK-positive on the DNA level (PCR and NGS), but ALK-negative on the protein level (IHC D5F3), and the patient had primary drug resistance to crizotinib. By using the NGS and analysis of the fusion gene sequence, the fusion was found to be a null fusion that could not translate the kinase-active ALK fusion protein (45). In addition, some uncommon fusions generate the canonical EML4-ALK fusion transcript alone at the RNA level, probably because the intron region of the rare gene was spliced out during mRNA maturation. In this case, because of the limited availability of the patient samples, we were unable to perform the RNA-seq validation for the rare ALK fusion. However, the response of the patient to ALK-TKIs strongly suggested the intact reading frame of the rearrangement. In addition, the spliced isoform still retained the key intact kinase domain for the production of the active chimeric protein. Thus, since DNA sequencing fails to provide direct evidence of functional fusion outcomes, orthogonal assays that can be verified at the RNA or protein level are necessary for accurately guiding the optimal treatment.

In conclusion, the first clinical evidence of the efficacy of ALK-TKI in a NSCLC patient harboring MRPS9-ALK fusion is reported, which provides a certain therapeutic reference for the patients with such gene alterations. Adequate utilization of different assay techniques will disclose more comprehensive mechanisms of pathogenesis and progression of the disease and treatment resistance, and furthermore better serve clinical decision-making. However, there are non‐negligible difficulties in the real world. What can be done is to make optimal choices with the conscientious, explicit, and judicious use of the best current evidence.

## Data Availability Statement

The original contributions presented in the study are included in the article/supplementary material. Further inquiries can be directed to the corresponding author.

## Author Contributions

HZ: conception, design and manuscript review. BX and JX: manuscript writing and revision. GZ: analysis of original material and data. YG: literature research and discussion. All authors contributed to the article and approved the submitted version.

## Funding

The authors are grateful for the financial grants from the National Natural Science Foundation of China (Grant No:81973805); the Zhejiang Provincial TCM Science and Technology Project (Grant No: 2015ZA088); and the Zhejiang Provincial Project for the key discipline of traditional Chinese Medicine (Yong GUO, no, 2017-XK-A09, http://www.zjwjw.gov.cn/).

## Conflict of Interest

The authors declare that the research was conducted in the absence of any commercial or financial relationships that could be construed as a potential conflict of interest.
